# Improving the Properties of Gelatin-Based Films by Incorporation of “Pitanga” Leaf Extract and Crystalline Nanocellulose

**DOI:** 10.3390/foods13101480

**Published:** 2024-05-10

**Authors:** Larissa Tessaro, Ana Gabrielle R. Pereira, Milena Martelli-Tosi, Paulo José do Amaral Sobral

**Affiliations:** 1 Department of Food Engineering, Faculty of Animal Science and Food Engineering, University of São Paulo, Av Duque de Caxias Norte, 225, Pirassununga 13635-900, SP, Brazil; ana_pereira@usp.br (A.G.R.P.); mmartelli@usp.br (M.M.-T.); pjsobral@usp.br (P.J.d.A.S.); 2 Food Research Center (FoRC), University of São Paulo, Rua do Lago, 250, Semi-Industrial Building Block, São Paulo 05508-080, SP, Brazil

**Keywords:** nanocomposite, active films, biopolymer, plant extract, agricultural by-product, physical properties

## Abstract

Biopolymer-based films can be activated by the incorporation of active compounds into their matrix. Plant extracts are rich in phenolic compounds, which have antimicrobial and/or antioxidant properties. The aim of this study was to produce gelatin-based active films and nanocomposite films incorporated with “pitanga” (*Eugenia uniflora* L.) leaf extract (PLE) and/or crystalline nanocellulose extracted from soybean straw (CN), and to study the physicochemical, functional, microstructural, thermal, UV/Vis light barrier, and antioxidant properties of these materials. PLE enhanced some film properties, such as tensile strength (from 30.2 MPa to 40.6 MPa), elastic modulus (from 9.3 MPa to 11.3 MPa), the UV/Vis light barrier, and antioxidant activity, in addition to affecting the microstructural, surface, and color properties. These improvements were even more significant in nanocomposites simultaneously containing PLE and CN (59.5 MPa for tensile strength and 15.1 MPa for elastic modulus), and these composites also had lower moisture content (12.2% compared to 13.5–14.4% for other treatments) and solubility in water (from 48.9% to 44.1%). These improvements may be the result of interactions that occur between PLE’s polyphenols and gelatin, mainly in the presence of CN, probably due to the formation of a stable PLE–CN–gelatin complex. These results are relevant for the food packaging sector, as the activated nanocomposite films exhibited enhanced active, barrier, and mechanical properties due to the presence of PLE and CN, in addition to being entirely produced with sustainable components from natural and renewable sources.

## 1. Introduction

The food and beverage industry is notorious for its extensive use of non-biodegradable packaging materials, such as single-use plastics, which often end up polluting ecosystems and harming wildlife. Additionally, large-scale agricultural practices associated with this industry can contribute to deforestation, soil degradation, and water pollution through the heavy use of pesticides, fertilizers, and irrigation. The disposal of food waste further exacerbates environmental concerns, as organic matter decomposes in landfills, emitting methane, a potent greenhouse gas. Consequently, addressing these issues requires comprehensive strategies to minimize waste generation, promote sustainable packaging alternatives, and encourage responsible production and consumption practices within the industry [1]. Specifically, an alternative way to avoid or reduce these problems is to replace plastic packaging with more eco-friendly materials, such as biopolymer-based films [2].

Edible and flexible biopolymer-based films can be used as food packaging, as they offer greater safety and can also extend shelf life. This technology emerged as an alternative to the use of conventional packaging, as it has characteristics such as biodegradability, biocompatibility, and is produced with natural and renewable components [3], and therefore can be considered eco-friendly and safe at the generally recognized as safe (GRAS) level [4].

Biopolymers such as proteins, such as gelatin and chitosan, and polysaccharides, such as starch, are generally used for the development of biopolymer-based films [5,6,7]. Gelatin, a versatile material utilized in the food industry, offers enhanced elasticity, stability, and consistency to food products. Moreover, it boasts exceptional barrier properties, permeability, and is characterized by its biocompatibility, biodegradability, and non-toxicity [8]. These attributes make gelatin an ideal candidate for coatings or films production, effectively prolonging the shelf life of food items. Derived from collagen, gelatin can be obtained through either alkaline or acid processes, resulting in type B or type A gelatin, respectively. Furthermore, gelatin can be sourced from mammals or marine origins, and is produced all over whole world [9], adding to its diverse applications within the food sector [8]. Gelatin-based films (or films based on other biopolymers) incorporated with active components constitute active films. Depending on the active component incorporated, active films, when applied as food packaging, can perform enhanced functions that help preserve food, extending shelf life and allowing the incorporation of lower amounts of preservatives into products.

The antioxidant and antimicrobial activities of plant extracts make them particularly interesting components for biopolymer-film activation, reducing the use of synthetic food preservatives [6]. Pomegranate peel extract [4], *Malva sylvestris* extract [10], hawthorn fruit extract [9], and “pitanga” leaf extract [6,11,12], among others, have been used to activate films based on gelatin or gelatin blended with other biopolymers. In addition to providing antioxidant and/or antimicrobial activity to the gelatin films, the plant extracts can also improve some properties of the films by chemical modification of the protein by active compounds.

**Figure 1:**
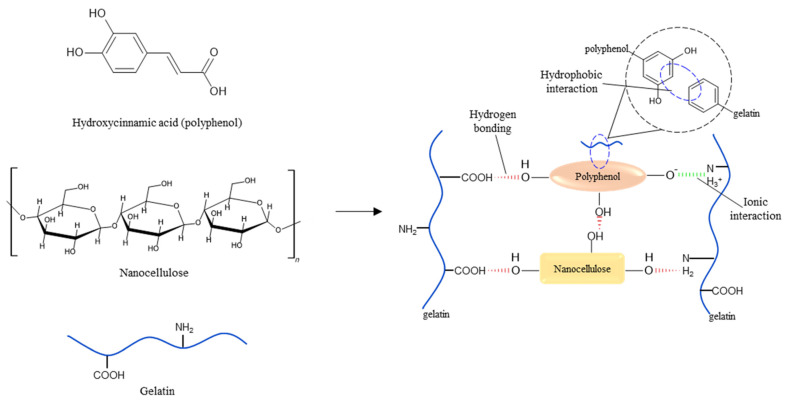
Non-covalent interactions that can occur between gelatin, polyphenols, and nanocelluloses. Source: Modified from Quan et al. [13]. Reproduced with permission from Tran Hong Quan, Soottawat Benjakul, Thanasak Saeleaw, Amjad Khansaheb Balange, and Sajid Maqsood. Trends in Food Science and Technology; published by Elsevier, 2019.

Plant extracts are substances rich in polyphenols and other active substances. Polyphenols are considered natural antioxidants, antimicrobials, and/or anti-inflammatories, as they have at least one aromatic ring linked to one or more hydroxyl groups, which allows them to prevent or delay oxidative degradation induced by reactive oxygen species [13]. Because of their structure, polyphenols can interact with gelatin via covalent and non-covalent interactions, with the latter (ionic, hydrophobic, and hydrogen bonds) (Figure 1) being the most common [13]. In fact, Vargas-Torrico et al. [4] and Yan et al. [9] have reported improvements in the properties of gelatin-based films incorporated with plant extracts, mainly in water sensitivity and mechanical properties, possibly caused by polyphenol–gelatin interactions.

In particular, “pitanga” (*Eugenia uniflora* L.) leaf extract (PLE) has found potential application in active films. “Pitanga” is a native Brazilian tree, which has edible fruits, also called “pitanga” and “Brazilian cherry”, and dark green leaves, widely used to produce teas that assist in folk medicine in the treatment of various diseases and symptoms [14]. More than 160 polyphenols were identified in the PLE, with hydroxycinnamic acids being the most abundant, followed by tyrosols, among other classes such as hydroxycoumarins, alkylmethoxyphenols, flavonoids, and flavones [15]. This wide variety of polyphenols gives PLE high antioxidant and antimicrobial activities, and it can then enhance active films for better biological activities and, possibly, physicochemical and mechanical properties of films. Gelatin-based packaging activated with PLE has been shown to reduce lipid oxidation and bacterial counts in dried-cured Coppa throughout the storage period [16].

Despite the improvements that plant extracts can provide to gelatin-based active films, they still present poor mechanical and water sensitivity properties, which limits their applications in areas such as food packaging. Just as gelatin is versatile and can incorporate different active compounds, it can also be combined with reinforcing fillers, such as nanocellulose, to form nanocomposite films to overcome these limitations [10]. Nanocellulose is extracted from cellulose in a nanostructured form. Cellulose of vegetable origin can come from different materials, such as waste generated by agricultural production, including that of soybean straw, residue from the threshing of soybeans, which is one of the largest commodities from Brazil [17].

Nanocellulose has characteristics such as high crystallinity, biocompatibility, biodegradability, and low toxicity [18], which makes it an interesting reinforcement material for biopolymer-based films, as it can improve the mechanical and thermal properties and water sensitivity of films [7]. Because of its structure, nanocellulose can interact with biopolymers such as gelatin, and with compounds such as polyphenols, through hydrogen bonding (Figure 1). In gelatin-based active nanocomposite films, for example, this means that a more stable gelatin–polyphenol–nanocellulose complex can be formed, improving the performance of these films’ properties [2,10].

In a previous study [11], we developed gelatin-based nanocomposite films, activated with PLE and encapsulated in a double emulsion, with improved properties. The effect of PLE together with NC on the properties of these materials was not clear, as it was encapsulated. Based on our knowledge, the effect of the interaction between PLE and nanocellulose on the properties of gelatin-based films and nanocomposite films has not been reported. In this study, we hypothesized that the simultaneous presence of PLE, rich in polyphenols, and crystalline nanocellulose in the gelatin matrix can lead to the formation of complexes and stable interactions that, consequently, improve the performance of nanocomposite films for applications such as food packaging. Hence, the aim of this study was to produce gelatin-based active films and nanocomposite films incorporated with PLE and/or crystalline nanocellulose extracted from soybean straw, and to study the physicochemical, functional, microstructural, thermal, UV/Vis light barrier, and antioxidant properties of these materials.

## 2. Material and Methods

### 2.1. Material

The PLE was produced using “Pitanga” leaves collected in the city of Pirassununga-SP, Brazil (21°59′46″ S, 47°25′36″ W). Embrapa Soja (Londrina-PA, Brazil)-supplied soybean straw was used to extract CN. The reagents used in the production of PLE and CN (acetone P.A., sulfuric acid P.A., ethanol P.A., sodium hydroxide P.A., hydrogen peroxide, and magnesium sulfate heptahydrate) were purchased from Química Dinâmica Contemporânea LTDA (São Paulo-SP, Brazil).

To produce the films and nanocomposite films, Gelnex (Itá-SC, Brazil) supplied type B bovine gelatin (bloom 225, average molecular weight 4–5 × 104 Da), and glycerol (95% purity) was purchased from Labsynth^®^ (São Paulo-SP, Brazil).

For antioxidant analysis, the reagents 2,2′-azino-bis(3-ethylbenzothiazoline-6-sulphonic acid) (ABTS), 6-hydrox-2,5,7,8-tetramethylchorman-2-carbxylic acid (Trolox), (2-pyridyl)-1,3,5-triazine (TPTZ), gallic acid, and iron (III) chloride hexahydrate were purchased from Sigma-Aldrich (St. Louis, MO, USA). Folin–Ciocalteu reagent, potassium persulfate, sodium acetate trihydrate, and sodium carbonate were purchased from Merck (Darmstadt, Germany).

### 2.2. Production of the “Pitanga” Leaf Extract

The PLE was produced according to methodology described by Tessaro et al. [12]. “Pitanga” leaves were collected, selected based on color and integrity (dark green and without damage), sanitized with water with detergent and distilled water, and soaked in sodium hypochlorite (0.1% *w*/*v*) for 15 min. Then, the sanitized leaves were dehydrated at 42 °C for 72 h (forced-air circulation drying oven, MA035, Marconi, Piracicaba-SP, Brazil). The dried leaves were ground in a commercial blender and sieved (48 mesh) to make the powder uniform. The powdered leaves were then dispersed in a 60% hydroethanolic solution (1 g powdered leaves/10 mL solution), and the formed dispersion was homogenized and subjected to ultrasonic extraction for 40 min (Ultrasound, MaxiClean 1400A, Unique, Indaiatuba-SP, Brazil), and then heated until 80 °C for 30 min under stirring (Magnetic stirrer integrated with temperature digital control, AA-2050, Gehaka, São Paulo-SP, Brazil). The formed PLE was filtered through a paper filter (Whatman n°1), rotary evaporated at 42 °C for 5 h (Rotary Evaporator Systems, TE 211, Tecnal, Piracicaba-SP, Brazil), and freeze-dried in a freeze-dryer (FD 1.0-60E, Heto-Holten A/S, Allerod, Denmark). The freeze-dried PLE was sieved (48 mesh) and stored protected from light in a freezer (−18 °C).

### 2.3. Production of the Crystalline Nanocelluloses

The CNs were extract from soybean straw (SS), according to methodology described by Martelli-Tosi et al. [17] with slight modifications. Init1ially, the SS (stem and 11pod) was separated, washed with running water, dried at 50 °C for 24 h in a forced-air circulation drying oven, ground in a knife mill (SL31, Solab, Piracicaba-SP, Brazil) and sieved (35 mesh). The ground SS was subjected to an alkaline pre-treatment with a 17.5% (*m*/*v*) sodium chlorite solution (100 g SS/1 L solution) under vigorous agitation at room temperature for 15 h (Mechanical stirrer, TE 039, Tecnal, Piracicaba-SP, Brazil). The pre-treated SS was washed with water and finally distilled water until it reached a neutral pH, using a set of sieves (200 and 400 mesh). Then, the pre-treated SS was bleached with 1 L of bleaching solution (4% hydrogen peroxide, 0.3% magnesium sulfate heptahydrate, and 2% sodium chlorite, *w*/*v*) under vigorous stirring at 90 °C for 3 h using a magnetic stirrer integrated with a digital temperature control.

The bleached SS was washed using a set of sieves (200 and 400 mesh) with water until neutral pH, followed by washings with distilled water, ethanol PA, and acetone PA, consecutively, and then dried at 50 °C for 4 days in a forced-air circulation drying oven. The bleached and dried SS was then ground in a commercial blender, sieved (28 mesh), and submitted to an acid hydrolysis with 64% (*w*/*v*) sulfuric acid solution (1 g SS/30 mL solution) under stirring at 65 °C for 40 min in a magnetic stirrer integrated with a digital temperature control. Finally, the acid hydrolysis reaction was stopped by diluting the dispersion with distilled water 10 times, and letting the CN and SS decant for 24 h. The decanted material was dialyzed (cellulose membrane) with tap water until neutral pH, and later homogenized in a rotor stator homogenizer at 14,000 rpm for 5 min (Ultraturrax^®^ IKA T25, Labotechnki, Staufen, Germany), plus 3 min of 550 W probe-type sonication at 50% amplitude (SFX550, Branson Ultrasonics Corp., Danbury, CT, USA). The formed CN suspension was freeze-dried and stored at room temperature.

### 2.4. Production of Gelatin-Based Films

The films (F) and nanocomposite films (N) were developed using the casting method, which consisted of drying the respective film-forming solution (FFS) according to Tessaro et al. [11,12]. To prepare the film-forming solutions of F, gelatin (4 g/100 g FFS) was hydrated in distilled water for 30 min at room temperature, and solubilized at 55 °C for 10 min, using a thermostatic bath (MA-184/20, Marconi, Piracicaba-SP, Brazil). Then, glycerol (25 g/100 g gelatin) was added to FFSs under moderate magnetic stirring (AA-2050, Gehaka, São Paulo-SP, Brazil). To produce film-forming suspension of N, the freeze-dried CN was added during the hydration of gelatin in water, at a concentration of 4.5 g/100 g gelatin. To produce the F and N film-forming solutions, PLE was subsequently added, at a concentration of 0.25 g PLE/100 g of gelatin, also under moderate magnetic stirring.

All FFSs were deposited on acrylic plates (12 × 12 cm) and dehydrated at 30 °C for 24 h (air-forced circulation drying oven, MA035, Marconi, Piracicaba-SP, Brazil). Before characterization, F and N were conditioned for 7 days in desiccators containing a saturated NaBr solution, whose relative humidity (RH) was 58%, at 25 °C. For the scanning electron microscopy (SEM), atomic force microscopy (AFM), and Fourier transformed infrared spectroscopy (FTIR) analyses, the F and N had been previously conditioned in silica gel (RH = 0%) for 15 days at 25 °C.

Therefore, four treatments were produced: control and active films (F-C and F-PLE, respectively) and control and active nanocomposite films (N-C and N-PLE, respectively).

### 2.5. Characterization of the Gelatin-Based Films and Nanocomposite Films

#### 2.5.1. Visual Aspect and Thickness

The appearance, homogeneity, and uniformity of the films were visually observed and described as their visual aspect [12].

Ten thickness measurements at random points were carried out on the film surfaces with a digital micrometer (±0.001 mm; Mitutoyo, Tokyo, Japan). The thickness of the films was calculated as the average of these measurements [12].

#### 2.5.2. Moisture Content

Film samples with known weights were dried at 105 °C for 24 h in a forced-air circulation drying oven, and moisture content was expressed as g water/100 g wet film [19].

#### 2.5.3. Solubility in Water

Film samples (2 cm diameter and known weight) were added to 50 mL of distilled water and shaken at 25 rpm and 25 °C for 24 h in an orbital shaker (MA141, Marconi, Piracicaba-SP, Brazil). The non-solubilized part of the films was filtered and dried at 105 °C for 24 h in a forced-air circulation drying oven. Solubility in water (SW) was calculated as the difference between initial and end film weights, in dry basis [19].

#### 2.5.4. Water Vapor Permeability

The water vapor permeability (WVP) of films was determined using the E-96-E80 standard test [20] modified by Gontard et al. [19]. Film samples of 30 mm diameter were fixed in aluminum permeation cells containing silica gel inside (RH = 0%) and with a permeation area of 31.17 cm^2^. These systems were placed in desiccators containing distilled water (RH = 100% and vapor pressure = 3.2691 kPa), and the permeation mass gain for each cell was noted at 24 h intervals for 7 days. The WVP (g·mm/m·h·kPa) of the films was calculated using Equation (1), where Δ*g*/Δ*t* is the rate of weight change (g/h), *x* is the thickness film (mm), *A* is the permeation area (cm^2^), and Δ*P*, is the partial pressure difference across the films (kPa).

(1) $$WVP=\frac{{∆}_{g}}{{∆}_{t}}\left(\frac{x}{A∆P}\right)$$

#### 2.5.5. Water Contact Angle

The water contact angle (WCA) with the air-side surface of the films was determined using an optical tensiometer (Attension Theta lite, KSV Instruments, Helsinki, Finland) equipped with OneAttension image analysis software (Version 4.1.9.8). Film samples were fixed to the equipment support and a drop of Milli-Q water was deposited on the film’s air-side surfaces using a precision syringe. Images were recorded each second for 60 s, and the WCA values were obtained in the chosen time of 15 s [12].

#### 2.5.6. Scanning Electron Microscopy (SEM)

The air-side surface and the cryo-fractured cross-section of the films were analyzed in random positions using a Hitachi tabletop microscope-SEM (TM3000, Hitachi Ltd., Tokyo, Japan), operating at a voltage of 15 kV. For the analysis of the drying surface, the film samples (20 × 20 µm) were fixed on stubs without any prior preparation. For the cryo-fractured internal structure analysis, the film samples were cryo-fractured after freezing with liquid nitrogen [12].

#### 2.5.7. Atomic Force Microscopy (AFM)

The topography and roughness of the air-side surface of the films (20 × 20 µm) were analyzed using atomic force microscopy (AFM NT-MDT, Moscow, Russian) in semi-contact mode with a resonance frequency of 240 kHz, contact force of 11.8 N/m, and scan speed of 0.3 Hz. The average roughness (Ra) was calculated as the absolute value of height deviations using the Nova Px 3.2.5 Rev 1266 equipment software [12].

#### 2.5.8. Gloss

The gloss of films was determined using a glossimeter (NGL 20/60, Rhopoint, Bexhill on Sea-West) at an angle of 60°, according to the D523 standard test [21]. Ten measurements were made at random points on the films’ air-side surface, and the gloss was expressed as a gloss unit (GU).

#### 2.5.9. Color and Opacity

The color parameters (L*, a*, and b*) were obtained using a MiniScan colorimeter (MSEZ 1049, HunterLab, Reston, VA, USA) in reflectance mode (CIELab scale, illuminant/angle D65/10°, 30 mm opening), according to Tessaro et al. [12]. Total color difference (ΔE*) was calculated with Equation (2), where ΔL* = L*sample–L*standard (93.59), Δa* = a*sample–a*standard (−1.00), and Δb* = b*sample–b*standard (1.75).

(2) $${∆E}^{*}=\sqrt{({{∆L}^{*})}^{2}+({{∆a}^{*})}^{2}+({{∆b}^{*})}^{2}}$$

The opacity (*Y*) of the films was obtained using the same MiniScan colorimeter with the same parameters. The opacity was calculated using Equation (3), where *Yb* is the opacity of the films under the standard black plate, and *Yw* is their opacity under the standard white plate.

(3) $$Y=\frac{Y_{b}}{Y_{w}}$$

#### 2.5.10. UV/Vis Light Barrier

The UV/Vis light barrier properties of films were determined using a UV-Vis spectrophotometer (Lambda 35, Perkin-Elmer, Waltham, MA, USA) in transmittance mode and in the wavelength from 200 to 800 nm [12]. Film samples (10 × 40 mm) were fixed in the cuvette so that to light passed through the films.

#### 2.5.11. Fourier Transformed Infrared Spectroscopy

Fourier transformed infrared spectroscopy (FTIR) analyses were performed using a spectrophotometer (Spectrum-One, Perkin-Elmer, Waltham, MA, USA) equipped with a UATR accessory (universal attenuator total reflectance), according to Tessaro et al. [12]. No prior preparation of the films was necessary. FTIR spectra were obtained by performing 20 scans in the spectral range from 4000 to 650 cm^−1^, with a resolution of 4 cm, and analyzed with the Spectrum-One 5.3 software (Perkin-Elmer, Waltham, MA, USA).

#### 2.5.12. Thermal Properties

The thermal properties of the films were determined using a differential scanning calorimeter (DSC TA2010, TA Instruments, New Castle, DE, USA) [11]. The film samples, placed in hermetically sealed aluminum TA pans, were heated from −50 to 150 °C at 5 °C/min, twice, in an inert atmosphere (45 mL/min N_2_). An empty pan was used as a reference. Before both scans, the DSC cell was cooled using liquid nitrogen. The glass transition (Tg) and melting (Tm) temperatures and the melting enthalpy (ΔHm) were calculated directly from the thermal curves using the Universal Analysis V1.7F software (TA Instruments, New Castle, DE, USA).

#### 2.5.13. Mechanical Properties

The mechanical properties of the films were determined using D882/12 axial tension tests [22] in a texturometer (TA.XT2i, Stable Micro Systems, Surrey, England) at room temperature. Film samples (15 × 100 mm) were fixed on grips separated by 50 mm, and the test was carried out with a grips separation speed of 1 mm/s. The tensile strength and the elongation at break were obtained directly from the stress versus strain curve, and the elastic modulus was calculated from the slope of the linear part of the curve, using the equipment software (Exponent Line Express, v4.0.13.0).

#### 2.5.14. Folin-Ciocalteu Reagent Reducing Compounds and Antioxidant Activities

Before testing, the films were cut into small fragments and shaken in an orbital shaker at 50 rpm (MA141, Marconi, Piracicaba-SP, Brazil) for 12 h, protected from light, with 50% hydroethanolic solution. Supernatants were used for analyses.

The quantification of Folin–Ciocalteu reducing capacity (FCRC) was carried out using the methodology described by Singleton et al. [23]. Antioxidant activities were determined by the ABTS free radical capture method (ABTS^●+^ method) [24], and the ferric reduction antioxidant power method (FRAP method) [25,26]. The antioxidant activity according to the ABTS^●+^ and FRAP methods was expressed in mg Trolox equivalent (TE)/g of film, and the activity according to the CRFC was expressed in mg gallic acid equivalent (GAE)/g of film.

### 2.6. Statistical Analysis

All samples were produced in triplicate and analyses were performed with at least three measurements from each replicate. The results of the film and nanocomposite film characterizations were presented as mean ± standard deviation and were subjected to analysis of variance (ANOVA) and mean comparisons using Tukey’s test (α = 0.05), using the Statistica 7.0 software.

## 3. Results and Discussion

### 3.1. Visual Aspect and Thickness

**Figure 2:**
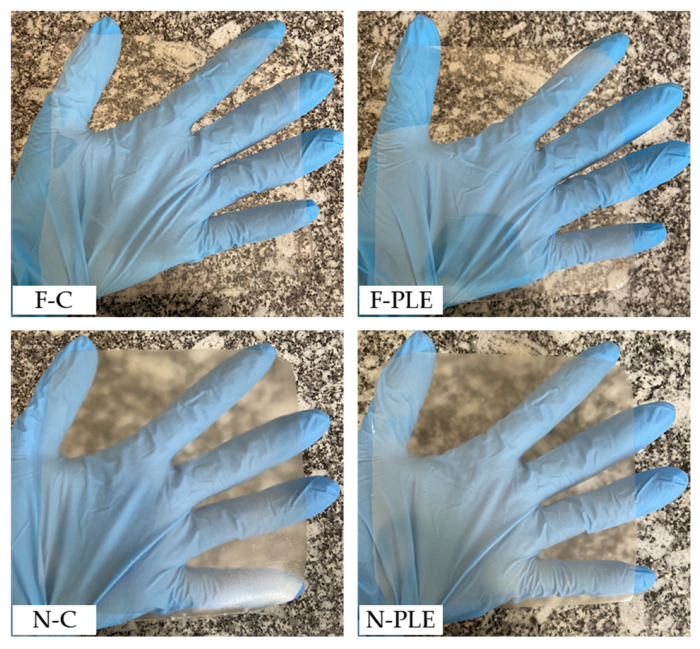
Examples of films (F) and nanocomposite films (N): control (C) and activated with “pitanga” leaf extract (PLE).

After drying, the films (F-C and F-PLE) were transparent and presented a homogeneous and uniform appearance, without visible bubbles (Figure 2). The nanocomposite films (N-C and N-PLE) were translucent and presented a less homogeneous appearance, possibly due to some CN agglomerates; however, they remained without bubbles or fractured regions (Figure 2).

Regarding thickness, no difference (*p* > 0.05) was observed between all samples (Table 1), indicating that the control of the mass of FFSs deposited on the plates (1.25 g dry matter/plate) was efficient. This achievement is important because thickness can influence some physical properties of biopolymer-based films [27].

### 3.2. Moisture Content

The moisture content can influence some physical properties of biopolymer-based films. Thus, it is important to know the moisture content of films, which is linked with their hygroscopicity [27]. The effect of adding PLE on moisture content was different for films and nanocomposite films (Table 1).

F-PLE showed higher moisture content than F-C, while the opposite effect was observed between N-C and N-PLE (*p* < 0.05). The increase in moisture content in F-PLE can be explained by the interactions that occurred between the PLE’s compounds and gelatin, increasing the affinity of the film for water molecules. In a previous study, the same occurred for gelatin-based films with haskap berries extract [5]. For nanocomposite films, PLE caused a decrease in moisture content, possibly due to the ability of PLE to interact with CN chains. According to Alzate-Arbeláez et al. [18], extracts rich in polyphenols form stable complexes with CN, due to the various interactions that can occur between them. More specifically, the -OH groups of PLE bind to the -OH groups of CN and gelatin through hydrogen bonds, reducing the hydrophilic groups available to interact with water molecules [9]. This decrease in moisture content was also observed in gelatin/chitosan nanocomposite films incorporated with anthocyanin-rich hawthorn fruit extract [9].

In the case of the effect of CN addition, F-C and N-C did not present different moisture content (*p* > 0.05), while N-PLE presented lower moisture content than the F-PLE film (*p* < 0.05). As previously explained, for the N-PLE film, the simultaneous presence of PLE and CN gave rise to stable complexes within the biopolymeric matrix (Figure 1), which interacted with each other and with the gelatin, possibly decreasing water absorption when compared to F-PLE, incorporated only with PLE.

### 3.3. Solubility in Water (SW)

The PLE increased (*p* < 0.05) the solubility in water of F-PLE in relation to F-C, but did not change (*p* > 0.05) the SW between N-PLE and N-C (Table 1). In the case of N-PLE, the presence of PLE did not affect the solubility in water, possibly because PLE can interact with CN structures [9], while in F-PLE, it can increase the hydrophilicity of the film and, consequently, its SW. Luciano et al. [6], who studied bi-layer gelatin-based films incorporated with PLE, reported that the addition of PLE did not affect the solubility in water of the films.

Regarding the effect of CN, a decrease in the solubility in water of N-PLE was noted in relation to the F-PLE film (*p* < 0.05), but no difference was observed between F-C and N-C (*p* > 0.05), thus as observed for moisture content. Likewise, this behavior has been observed for gelatin/carboxymethylcellulose incorporated with pomegranate peel extract [4]. Although CN presents greater interfacial bonding, forming networks, which theoretically hinders the diffusion of water through the films [7], this effect was not observed between the film and nanocomposite film controls.

### 3.4. Water Vapor Permeability (WVP)

Among all of the treatments studied, there was no difference (*p* > 0.05) in relation to WVP, that is, the effect of adding NC and/or PLE did not change the WVP of the films and nanocomposite films (Table 1).

Regarding the effect of PLE, the amount of PLE added was not able to change and cause differences between the WVP of films and nanocomposite films, as observed by Luciano et al. [6]. Because they were gelatin-based, the films and nanocomposite films had great affinity and solubility in water, justifying the high WVP presented by them [12], even charged with CN. The WVP values were similar to those determined by other authors working on gelatin-based films with PLE or nisin (3.1 to 4.0 × 10^−1^ g·mm/m^2^·h·kPa) [6] and carboxymethyl cellulose and pomegranate peel extract (around 2 × 10^−1^ g·mm/m^2^·h·kPa) [4]. In terms of application, all of these films can be considered very permeable to water vapor [11].

### 3.5. Water Contact Angle (WCA)

WCA results provide information regarding the hydrophobicity of the film surface, and several factors can interfere with this parameter, such as surface roughness and the hydrophobicity of the components of the biopolymeric matrix. If the surface of the film is rougher, the contact surface of the water with the film will consequently be larger, causing its contact angle to increase [12].

All of the films and nanocomposite films were considered to have a hydrophobic air-side surface, with θ > 65° [28] (Table 2). When analyzing the effect of PLE, it was observed that F-C presented higher WCA than F-PLE (*p* < 0.05), which has also been reported for gelatin/inulin nanocomposite films incorporated with crystalline nanocellulose and *Malva sylvestris* extract [10]. PLE’s hydrophilic molecules can increase the hydrophilicity of the film, increasing its affinity for water [5]. Although the average roughness of the air-side surface of F-PLE was higher than that of F-C (Table 2), this was not the main factor that affected the WCA of F-PLE, since both films (F-C and F-PLE), showed very low average roughness. For nanocomposite films, PLE did not affect WCA (*p* > 0.05), nor did it affect the average roughness (Table 2). In other words, the addition of PLE did not affect the contact surface of the N-PLE film with water.

The addition of CN decreased the WCA of N-C compared to that of F-C (*p* < 0.05). The same behavior was observed by Pereda et al. [29] for sodium caseinate-based films and nanocomposite films, which was related to increased film roughness. Comparing F-PLE and N-PLE, no difference was observed in WCA (*p* > 0.05).

### 3.6. Scanning Electron Microscopy (SEM)

**Figure 3:**
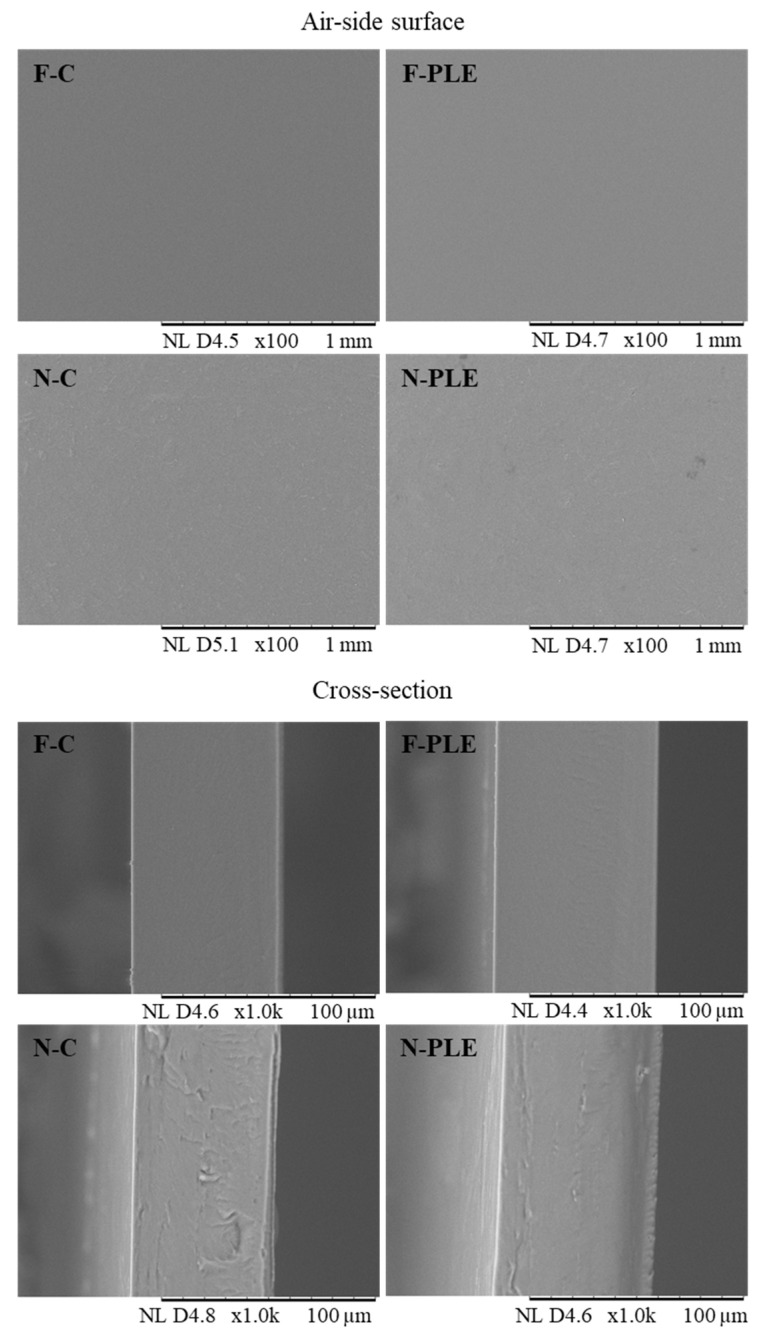
Electronic scanning micrographs of the air-side surfaces and cross-section of films (F) and nanocomposite films (N), control (C) and activated with “pitanga” leaf extract (PLE).

SEM was used to analyze the microstructure of the samples. No differences were observed between F-C and F-PLE, both in the air-side surfaces and in the cross sections (Figure 3). These films presented smooth, continuous, and homogeneous surfaces, without any phase separation, demonstrating a good incorporation of PLE into the biopolymeric matrix, as observed for bi-layer gelatin films incorporated with PLE and/or nisin [6] and gelatin-based films with haskap berries extract [5].

For N-C and N-PLE, a certain heterogeneity and discontinuity was noted on the air-side surfaces and in the cross sections (Figure 3) of both, possibly caused by CN agglomerations, as has been previously observed for gelatin/inulin-based nanocomposite films containing CN and *Malva sylvestris* extract [10]. However, no fracture or phase separation was observed, indicating that there was good incorporation of CN into the biopolymeric matrices. Furthermore, in N-PLE, the heterogeneity and possible agglomerations of CN were less evident than in N-C, especially in the cross section, which may indicate that there was interaction between the PLE and the CN that improved the incorporation of the CN in the biopolymeric matrix [10].

### 3.7. Atomic Force Microscopy (AFM)

**Figure 4:**
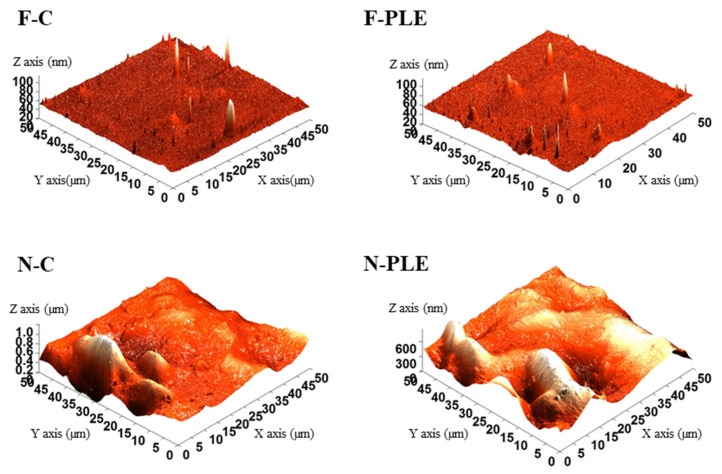
3D atomic force micrographs of the air-side surfaces of films (F) and nanocomposite films (N), control (C) and activated with “pitanga” leaf extract (PLE).

The results obtained by AFM analysis (Figure 4) showed that the films presented smoother topographies and small peaks, almost insignificant compared to the large peaks and valleys of the nanocomposite films. These observations corroborated the results presented in the SEM analysis (Figure 3). Similar results have previously been obtained for gelatin-based nanocomposite films with CN and PLE encapsulated in double emulsion [11].

Given the average roughness (Ra), obtained from the analysis of the topography of the samples, the effect of adding PLE and NC to the films and nanocomposite films was studied. Regarding the effect of PLE, F-PLE showed higher average roughness than F-C (*p* < 0.05) (Table 2), possibly because the incorporation of PLE caused slight accumulations, causing rougher regions. Similar observations were presented by Luciano et al. [6]. For N-C and N-PLE, there were no differences in Ra (*p* > 0.05). In this case, PLE did not increase, at least not detectably, the roughest regions already caused by CN.

Regarding the effect of adding CN, both between F-C and N-C, and between F-PLE and N-PLE, the Ra increased in the order of approximately 20,000 times (Table 2). CNs possess a nanoscale diameter, although their length can be in the range of submicrometers. The dispersion of these fibers throughout the bulk material may extend to the surface, leading to increased surface roughness. Additionally, structures tend to agglomerate, increasing the Ra of the nanocomposite films. High Ra was also found by Tessaro et al. [11] for gelatin-based nanocomposite films.

### 3.8. Gloss

The addition of PLE did not affect (*p* > 0.05) the gloss of F-PLE compared to the gloss of F-C, nor the gloss of N-PLE compared to the gloss of N-C (Table 2). The good incorporation of PLE into the biopolymeric matrices had little effect on the air-side surface roughness of the active films and nanocomposite films compared to their respective controls. This is certainly why the gloss was also not affected by the incorporation of PLE. The same behavior was observed by Luciano et al. [6] in their studies on bi-layer gelatin films with PLE. Tessaro et al. [11] observed a decrease in the gloss of films when studying gelatin-based films with CN and with or without the addition of PLE encapsulated in a double emulsion, possibly as an effect of the presence of the double emulsion, and not of the PLE itself.

The addition of CN decreased (*p* < 0.05) the gloss of N-C and N-PLE compared to F-C and F-PLE, respectively (Table 2), as the presence of CN can cause nanocomposite films with less polished and shiny air-side surfaces than the respective films, due to non-uniform distribution of nanoparticles or even agglomerations [7]. In fact, the air-side surface of the nanocomposite films was much rougher than that of the films, as already discussed. The same result was reported by Pelissari et al. [7] when studying nanocomposite films based on banana starch reinforced with cellulose nanofibers.

The film gloss is a surface property that is directly related to the morphology and roughness of the air-side surface of the films, that is, the degree of surface polishing. A negative linear dependence was observed in this case (Gloss = −16.0 Ra + 212.7, R^2^ = 0.966). Similar behavior was observed by Luciano et al. [6].

### 3.9. Color and Opacity

Color is an important property that affects the physical appearance of films and consequently consumer acceptance [4]. PLE has a greenish-yellow color (L* = 30.4, a* = 12.1, b* = 41.3), which may be associated with light dispersions from the phenolic compounds present in the PLE; therefore, it was expected that the color of the films and nanocomposite films would be affected by the PLE [12]. In fact, all of the color parameters (L*, a*, b* and ΔE*) showed differences (*p* < 0.05) when studying the effect of adding PLE on F-PLE and N-PLE compared to F-C and N-C, respectively (Table 2), except for the b* parameter for nanocomposite films, which showed no difference (*p* > 0.05).

For the L* parameter (brightness value), which varies from zero (white = light) to 100 (black = dark), the addition of PLE decreased its value (*p* < 0.05) in the film and nanocomposite film compared to their respective controls. The low L* value of the PLE may have caused the decrease in this parameter in the F-PLE and N-PLE.

For a*, b*, and ΔE*, the presence of PLE caused an increase in the values of these parameters in F-PLE and N-PLE. As expected, the color parameters of the films and nanocomposite films were influenced by the color parameters of the PLE, which has positive values for the a* and b* parameters, that is, it presents a reddish and yellowish color, respectively. Therefore, F-PLE and N-PLE showed stronger reddish and yellowish colors than their controls (F-C and N-C, respectively). The total color difference of F-PLE and N-PLE (evaluated by the ΔE* parameter) was also greater than that of F-C and N-C, which indicates that the active film and nanocomposite film were more colorful than the controls. Luciano et al. [6] also reported differences in the color parameters of their films caused by PLE, but their differences presented a less reddish color than those studied in this study. Vargas-Torrico et al. [4] also reported changes in all color parameters of gelatin-carboxymethylcellulose films and nanocomposite films upon addition of pomegranate peel extract.

The incorporation of CN also resulted in alterations to the color parameters. For the parameters L* and a*, there was a decrease in them when comparing the nanocomposite films with their respective films (Table 2), indicating that the nanocomposite films were less clear and less greenish, respectively. For the parameters b* and ΔE*, the opposite occurred, indicating that the nanocomposite films were more yellowish and more colorful, respectively. In short, CN produced darker, less greenish and more yellowish nanocomposite films, probably due to the color of CN from soybean straw, which is slightly yellowish. The same trend in nanocomposite film coloring was observed by Pelissari et al. [7]. Nikoukheslat et al. [10] also reported changes in the color parameters of gelatin/inulin-based nanocomposite films by incorporation of CN and *Malva sylvestris* extract.

It is worth mentioning that although PLE and/or CN caused changes in the color parameters of films and nanocomposite films, all of the samples studied showed high luminosity (bright, with L* > 85), and values of a*, b*, and ΔE* closer to zero, indicating that the films and nanocomposite films produced were poorly colored.

The opacity was similar (*p* > 0.05) for F-C and F-PLE, and N-C and N-PLE (Table 2). Therefore, it can be considered that the amount of PLE used in the production of F-PLE and N-PLE did not generate insoluble regions that made it impossible for light to pass through them [6]. Luciano et al. [6] presented opacity values very close to those obtained in this study for F-C and F-PLE. N-C and N-PLE presented higher opacity than F-C and F-PLE, respectively. In other words, the nanocomposite films were less transparent, due to the solid particles of the CN and the strong interactions between the CN and gelatin, which decreased the passage of light through the nanocomposite films. The same behavior was observed by Pelissari et al. [7].

### 3.10. UV/Vis Light Barrier

**Figure 5:**
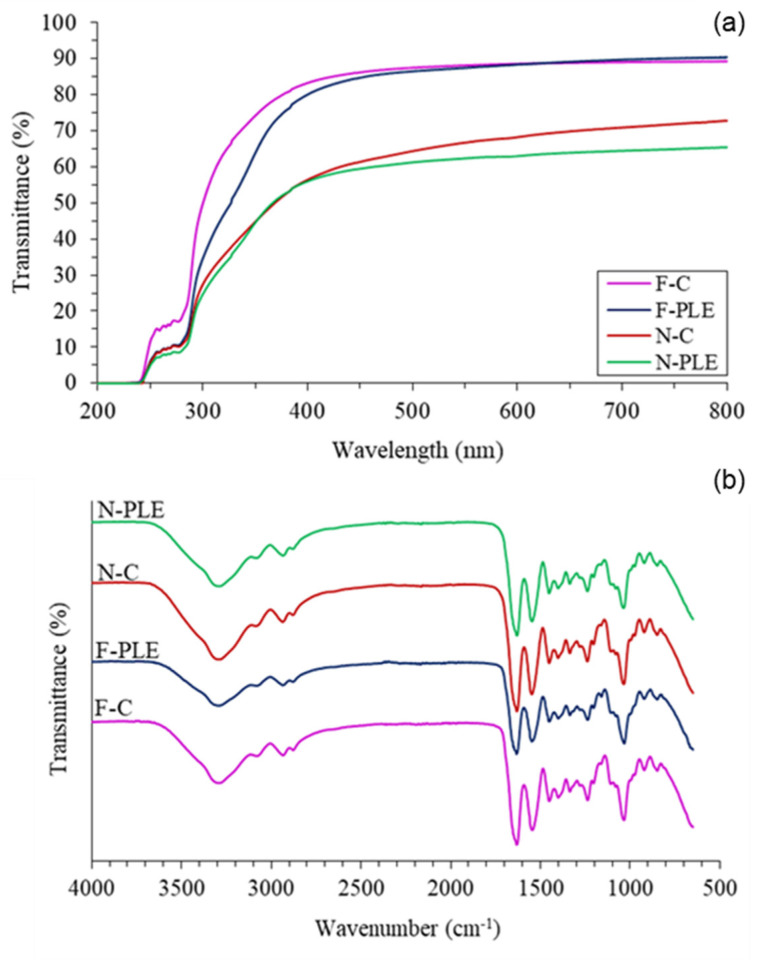
(**a**) UV/Vis spectra and (**b**) FTIR spectra of films (F) and nanocomposite films (N), control (C) and activated with “pitanga” leaf extract (PLE).

The UV/Vis light barrier is an important and desirable property for food packaging, as it can prevent and reduce lipid oxidation and nutrient loss induced by light incidence [5]. Between wavelengths of 200 and 300 nm (UV light), all samples showed low transmittance (<20%), that is, having high barrier properties to UV light (Figure 5a). The F-PLE, N-C, and N-PLE showed better performance in this region (~10% transmittance) than the F-C (~15% transmittance). Therefore, in the UV light region, the barrier properties varied as follows: N-PLE > N-C = F-PLE > F-C.

In the visible light region (above 300 nm), F-C showed higher transmittance than F-PLE up to 450 nm, while N-C and N-PLE showed the lowest transmittance in this same region, with no major differences between them. From 450 nm onwards, the transmittance of F-C and F-PLE was practically the same, with no notable differences, while N-PLE started to present lower transmittance than N-C. In general, the barrier properties in the visible light region were ordered as follows: N-PLE > N-C > F-PLE > F-C.

PLE is rich in polyphenols, which are capable of absorbing visible light, reducing the transmittance through films and nanocomposite films [4]. Other studies on gelatin films incorporating substances rich in polyphenols have also shown better barrier properties to UV/Vis light compared to control films [4,5,6]. For nanocomposite films, from 300 nm onwards, the transmittance was explicitly lower, that is, they had better barrier properties to visible light. This happened because NC makes light scatter more easily, reducing transmittance in nanocomposite films [29]. Similar results have been reported for potato starch-based nanocomposite films with bacterial nanocellulose and gallic acid [2].

The combination of the good visible light barrier properties of PLE and CN, as mentioned above, caused N-PLE to have lower transmittance and better efficiency. Furthermore, another important factor was the presence of amino acid residues containing aromatic rings in gelatin, which grants it excellent UV barrier properties [12].

### 3.11. Fourier-Transform Infrared Spectroscopy (FTIR)

The films and nanocomposite films showed characteristic bands of gelatin-based materials in the FTIR spectrum, with no difference observable due to the presence of PLE and/or CN (Figure 5b). The lack of differentiation between the samples may have been the result of similar chemical bonds that formed between the components, as well as in the gelatin–CN–PLE interaction, resulting in overlapping bands.

FTIR spectra showed bands at approximately 3287–3289 cm^−1^ (overlap of O-H and N-H stretching coupled with hydrogen bonding of amide A groups), probably due to hydrogen interactions between gelatin and glycerol (Figure 1), 2936–2935 cm^−1^ (=C–H and –NH^3+^ asymmetric stretching vibration, from amide B groups), 1636–1630 cm^−1^ (C-O stretching and hydrogen bonding coupled with COO, from amide-I groups) [4], due to ionic interactions between acyl groups of some amino acids of gelatin and functional groups of phenolic compounds of PLE (Figure 1); 1548 cm^−1^ (bending vibrations and N–H and C–N stretching, respectively, of the amide-II groups), 1451–1452 cm^−1^ (vibration of the -OH groups of the gelatin structure and/or CN and/or glycerol), and 1236 cm^−1^ (in-plane vibrations of the C–N and N–H groups, from amide-III groups, or CH_2_ vibrations from glycerol) [4].

### 3.12. Thermal Properties

**Figure 6:**
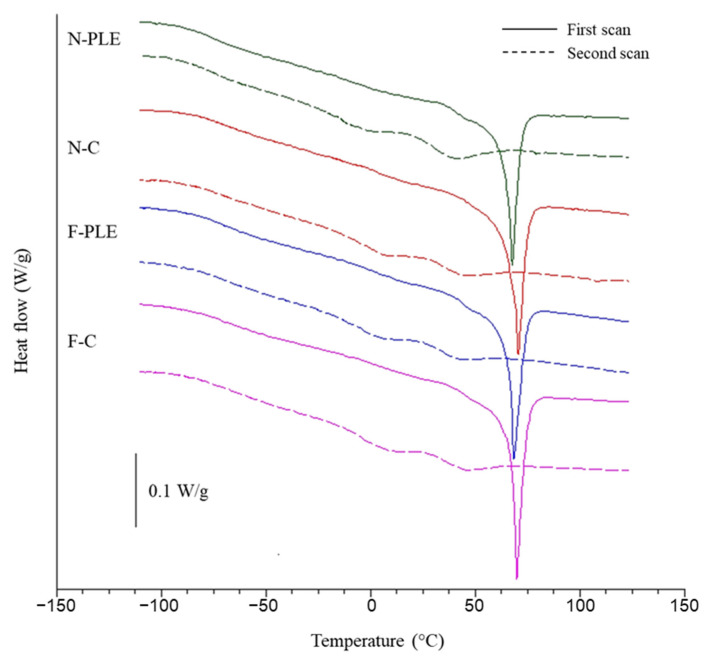
DSC thermal curves (Exo up) of films (F) and nanocomposite films (N), control (C) and activated with “pitanga” leaf extract (PLE).

The Tg, Tm, and ΔH_m_ of the biopolymer matrix were not affected (*p* > 0.05) by the addition of PLE nor CN (Table 3), that is, the added amount of these compounds was not sufficient to cause changes in thermal properties. Therefore, the thermograms (Figure 6) displayed by the films and nanocomposite films were similar.

For all of the samples studied, the thermograms of the first scan showed characteristics typical of partially crystalline materials. In the first scan, two Tgs were observed, Tg_1_ (around −70 °C) and Tg_2_ (around 45 °C), associated with the fractions rich in glycerol and gelatin, respectively. An endothermic peak was also observed in the first scan (Tm ≈ 69.5 °C), related to the melting of gelatin microcrystals [30], whose ΔH_m_ was ~17 J/g.

In the second scan, the thermograms obtained were characteristic of amorphous materials [6], showing only Tg1 (~73.5 °C) and Tg_2_ (~35 °C). In the case of Tg_2_, the value observed in the second scan was lower than the value obtained in the first scan (~45 °C), as the absence of gelatin microcrystals, which melted in the first scan, facilitated biopolymeric mobility and, consequently, decreased the Tg_2_. The Tg_1_ varied little between the two scans, as glycerol was found to be amorphous in both cases.

The values of thermal properties observed were similar to those of other authors who have studied gelatin-based films and nanocomposite films with the addition of plant extracts and/or CN [6,10].

### 3.13. Mechanical Properties

The insufficient performance of the mechanical properties of biopolymer-based films is one of the limitations that hinder their wide application in food packaging [2]. The incorporation of additives into biopolymer film matrices, such as reinforcing fillers, mainly seeks to improve the performance of mechanical properties and the applicability of these materials.

Regarding the effect of PLE, F-PLE and N-PLE presented higher tensile strength and elastic modulus (*p* < 0.05) than F-C and N-C, respectively (Table 4). The presence of PLE did not change (*p* > 0.05) the elongation at break of the films and nanocomposite films (Table 4). PLE’s polyphenols can interact with gelatin by (i) hydrogen bonding (-OH of the polyphenol interacts with -COOH and -NH_2_ of gelatin), (ii) hydrophobic interactions (non-polar aromatic ring of PLE interacts with hydrophobic amino acid residues of gelatin), and (iii) positively charged groups of the protein interacting with negatively charged hydroxyl groups of the PLE, forming a kind of crosslink [31], as can be observed in Figure 1, which increases the resistance and stiffness of the films (tensile strength and elastic modulus, respectively). Liu et al. [5] and Vargas-Torrico et al. [4] observed the same behavior in gelatin-based films with haskap berries extract, and gelatin/carboxymethylcellulose-based nanocomposite films with pomegranate peel extract, respectively.

As for the effect of CN, the films showed lower (*p* < 0.05) tensile strength and elongation at break than nanocomposite films (Table 4). N-PLE had a higher elastic modulus than F-PLE (*p* < 0.05), while F-C and N-C had the same elastic modulus (*p* > 0.05). The increase in the resistance of the nanocomposite films, and the decrease in flexibility, was possibly a consequence of the interactions that the CN made with the biopolymer matrix, forming an interconnected and more rigid network with reduced elongation of the biopolymer chains [32]. In the N-PLE case, the interactions between PLE and CN, simultaneously present in this nanocomposite film, may have resulted in the formation of a more stable matrix, increasing the rigidity of the film. Similar behaviors have been observed by Pelissari et al. [7].

Pereda et al. [29] also observed higher tensile strength and EM and lower elongation at break for sodium caseinate-based nanocomposite films with nanocellulose fibers. Likewise, in another study, potato starch-based nanocomposite films showed increased tensile strength and elastic modulus compared to films without bacterial nanocellulose [2]. This increase was more significant in the presence of gallic acid, and Almeida et al. [2] explained that hydrogen bonds possibly occur between the -OH groups of gallic acid, bacterial nanocellulose, and potato starch, forming a more stable and compact matrix.

Finally, the fact that N-PLE presented the highest tensile strength and elastic modulus, and lowest elongation at break, may also have been related to its moisture content, which was lower than in the other treatments. Reducing the moisture content in films and nanocomposite films can increase tensile strength and elastic modulus, and decrease elongation at break, as water has a plasticizing effect on hydrophilic components, such as gelatin [3].

### 3.14. Folin-Ciocalteu Reducing Capacity (FCRC) and Antioxidant Activity (AA)

The oxidative degradation of some foods, such as foods rich in lipids, is an important issue in the food industry, as undesirable products originating from the oxidation reaction can negatively alter the chemical and sensory characteristics of foods. The incorporation of antioxidant compounds into active food packaging is a very attractive alternative compared to adding them directly to food, due to the sensorial changes that can occur [2].

The films and nanocomposite films with PLE showed higher FCRC and AA (*p* < 0.05) than the respective controls (Table 5), as expected. The AA of the active film and nanocomposite film was due to the different classes of polyphenols present in PLE [14], which even at low concentrations, have an advantageous biological activity [33].

The presence of CN did not interfere with the FCRC of the nanocomposite films compared to the films (*p* > 0.05), but decreased the AA in N-PLE compared to F-PLE (*p* < 0.05). Some polyphenols can be adsorbed on CN surfaces, reducing the availability of these compounds, and hindering the extraction, which would explain the lower AA in N-PLE [11]. The greater AA provided by the FRAP method can be justified by the difference in the mechanism of action. The ABTS^●+^ method is based on the reduction of ABTS^●+^ by receiving hydrogen atoms or electrons from antioxidant compounds, and can be used for both hydrophilic and lipophilic substances [34]. On the other hand, the FRAP method is based on the reduction of ferric ions (Fe^3+^) to ferrous ions (Fe^2+^), which occurs in the presence of antioxidant compounds and, therefore, is an electron transfer method and can also be used to determine the AA of lipophilic and hydrophilic substances [35]. However, it presents some limitations, such as the fact that any compound with a redox potential lower than that of the Fe^3+^/Fe^2+^ pair can, in theory, reduce Fe^3+^ to Fe^2+^, thus generating overestimated results [35]. In the case of gelatin-based films, the higher AA in the FRAP method may be due to the activity of some functional groups of amino acids in bovine gelatin, such as proline and glycine, which can act as electron donors, providing an antioxidant effect [36]. Another factor that may influence the highest AA given by the FRAP method is the acidic pH of the assay, as phenolic compounds may eventually be more active in this media [34].

Gelatin-based films and nanocomposite films activated with plant extracts have also shown increased AA due to the presence of the extract rich in polyphenols [3,4,6].

## 4. Conclusions

The development of gelatin-based films and nanocomposite films incorporating polyphenol-rich extracts (PLE) and cellulose nanocrystals (CN) from soybean straw has demonstrated promising advancements in the field of active food packaging. The chemical interactions between gelatin, PLE polyphenols, and CN resulted in enhanced mechanical properties, UV/Vis light barrier capabilities, antioxidant activity, and surface properties in the films. Furthermore, the active nanocomposite films exhibited even greater improvements, including lower moisture content and solubility in water, compared to the active films. Despite these enhancements, both materials maintained desirable aesthetic qualities, being light, shiny, and translucent. These findings highlight the potential of utilizing sustainable components derived from natural and renewable sources in the production of active food packaging, offering both functional and environmental benefits for the food industry.

## Figures and Tables

**Table 1:** Physicochemical properties of films (F) and nanocomposite films (N), control (C) and activated with “pitanga” leaf extract (PLE) *.

Properties	Treatments	C	Active (PLE)
Thickness (mm)	F	0.083 ± 0.002 ^aA^	0.077 ± 0.004 ^aA^
	N	0.084 ± 0.006 ^aA^	0.077 ± 0.005 ^aA^
Moisture content (%)	F	13.5 ± 0.3 ^bA^	14.4 ± 0.4 ^aA^
	N	13.7 ± 0.2 ^aA^	12.2 ± 0.7 ^bB^
Solubility in water (%)	F	43.4 ± 0.4 ^bA^	48.9 ± 0.8 ^aA^
	N	42.9 ± 0.7 ^aA^	44.1 ± 1.0 ^aB^
WVP (×10^1^ mm·g/m^2^·h·kPa)	F	3.2 ± 0.3 ^aA^	3.0 ± 0.1 ^aA^
	N	3.6 ± 0.2 ^aA^	3.2 ± 0.1 ^aA^

* Means ± standard deviation (n = 3). Different lowercase letters on the same line and different uppercase letter in the same column indicate significant differences between means, according to Tukey’s test (*p* < 0.05). PLE: “Pitanga” leaf extract; WVP: Water vapor permeability.

**Table 2:** Air-side surface properties and color parameters of films (F) and nanocomposite films (N), control (C) and activated with “pitanga” leaf extract (PLE) **.

Properties	Treatments	C	Active (PLE)
Water contact angle (°)	F	80.9 ± 0.3 ^aA^	71.3 ± 0.9 ^bA^
	N	72.7 ± 0.3 ^aB^	72.1 ± 0.3 ^aA^
Average roughness (nm)	F	3.5 ± 0.3 ^bB^	5.2 ± 0.7 ^aB^
	N	(10.6 ± 0.8) × 10^4 aA^	(10.2 ± 1.1) × 10^4 aA^
Gloss (GU)	F	148 ± 3 ^aA^	142 ± 8 ^aA^
	N	49 ± 7 ^aB^	41 ± 1 ^aB^
L*	F	90.2 ± 0.1 ^aA^	88.8 ± 0.2 ^bA^
	N	88.7 ± 0.2 ^aB^	87.4 ± 0.1 ^bB^
a*	F	−1.3 ± 0.0 ^aA^	−0.9 ± 0.0 ^bA^
	N	−0.6 ± 0.0 ^aB^	−0.4 ± 0.0 ^bB^
b*	F	3.5 ± 0.1 ^bB^	4.0 ± 0.1 ^aB^
	N	6.9 ± 0.2 ^aA^	7.3 ± 0.3 ^aA^
ΔE*	F	4.1 ± 0.1 ^bB^	5.5 ± 0.2 ^aB^
	N	7.2 ± 0.1 ^bA^	8.5 ± 0.3 ^aA^
Opacity (%)	F	0.8 ± 0.1 ^aB^	0.8 ± 0.1 ^aB^
	N	2.0 ± 0.3 ^aA^	1.9 ± 0.2 ^aA^

** Means ± standard deviation (n = 3). Different lowercase letters on the same line and different uppercase letter in the same column indicate significant differences between means, according to Tukey’s test (*p* < 0.05). ΔE*: total difference of color.

**Table 3:** Thermal properties of films (F) and nanocomposite films (N), control (C) and activated with “pitanga” leaf extract (PLE) *.

Scan	Properties	Treatments	C	Active (PLE)
1st	T_g1_ (°C)	F	−75.7 ± 3.3 ^aB^	−70.9 ± 3.4 ^aA^
		N	−68.1 ± 1.2 ^aA^	−67.4 ± 1.6 ^aA^
	T_g2_ (°C)	F	44.4 ± 0.2 ^aA^	44.2 ± 1.4 ^aA^
		N	48.0 ± 3.1 ^aA^	44.1 ± 3.2 ^aA^
	T_m_ (°C)	F	69.6 ± 2.1 ^aA^	69.3 ± 1.0 ^aA^
		N	70.6 ± 0.5 ^aA^	69.4 ± 1.6 ^aA^
	ΔH (J/g)	F	18.3 ± 0.4 ^aA^	16.7 ± 1.2 ^aA^
		N	16.4 ± 1.5 ^aA^	17.3 ± 1.1 ^aA^
2nd	T_g1_ (°C)	F	−73.3 ± 3.7 ^aA^	−74.3 ± 4.0 ^aA^
		N	−72.4 ± 1.3 ^aA^	−73.8 ± 3.3 ^aA^
	T_g2_ (°C)	F	36.3 ± 1.9 ^aA^	34.8 ± 0.9 ^aA^
		N	35.5 ± 3.0 ^aA^	34.4 ± 3.3 ^aA^

* Means ± standard deviation (n = 3). Different lowercase letters on the same line and different uppercase letter in the same column indicate significant differences between means, according to Tukey’s test (*p* < 0.05). Tg: glass transitions temperatures; Tm: melting temperatures of gelatin microcrystals; ΔH: melting enthalpy of gelatin microcrystals.

**Table 4:** Mechanical properties of films (F) and nanocomposite films (N), control (C) and activated with “pitanga” leaf extract (PLE) *.

Properties	Treatments	C	Active (PLE)
Tensile strength (MPa)	F	30.2 ± 1.9 ^bB^	40.6 ± 5.3 ^aB^
	N	53.6 ± 2.6 ^bA^	59.5 ± 1.4 ^aA^
Elongation at break (%)	F	23.0 ± 1.3 ^aA^	26.7 ± 3.4 ^aA^
	N	12.7 ± 2.9 ^aB^	12.0 ± 1.2 ^aB^
Elastic modulus (MPa)	F	9.3 ± 0.9 ^bA^	11.3 ± 0.4 ^aB^
	N	10.9 ± 0.8 ^bA^	15.1 ± 0.4 ^aA^

* Means ± standard deviation (n = 15). Different lowercase letters on the same line and different uppercase letter in the same column indicate significant differences between means, according to Tukey’s test (*p* < 0.05).

**Table 5:** Folin–Ciocalteu reducing capacity (FCRC) and antioxidant activity of films (F) and nanocomposite films (N), control (C) and activated with “pitanga” leaf extract (PLE) *.

Properties	Treatments	C	Active (PLE)
FCRC (mg GAE/g sample)	F	0.0 ± 0.0 ^bA^	0.7 ± 0.0 ^aA^
	N	0.0 ± 0.0 ^bA^	0.7 ± 0.0 ^aA^
ABTS^•+^ (mg TE/g sample)	F	0.0 ± 0.0 ^bA^	3.2 ± 0.2 ^aA^
	N	0.0 ± 0.0 ^bA^	2.7 ± 0.2 ^aB^
FRAP (mg TE/g sample)	F	3.6 ± 0.3 ^bA^	10.6 ± 0.6 ^aA^
	N	3.2 ± 0.4 ^bA^	9.3 ± 0.4 ^aB^

* Means ± standard deviation (n = 3). Different lowercase letters on the same line and different uppercase letter in the same column indicate significant differences between means, according to Tukey’s test (*p* < 0.05). GAE: gallic acid equivalent. TE: Trolox equivalent.

## Data Availability

The original contributions presented in the study are included in the article, further inquiries can be directed to the corresponding author.
